# Beyond Fatigue: The Fatigue Assessment Scale as a Potential Indicator of Long COVID Severity

**DOI:** 10.3390/jcm15155878

**Published:** 2026-07-28

**Authors:** Giulia Del Duca, Marta Franco, Lorenzo Talamanca, Andrea Antinori, Marta Camici

**Affiliations:** 1 Clinical and Research Infectious Diseases Department, National Institute for Infectious Diseases Lazzaro Spallanzani IRCCS, 00149 Rome, Italy; giulia.delduca@inmi.it (G.D.D.); andrea.antinori@inmi.it (A.A.); 2 Endocrinology Clinical Unit, San Camillo Forlanini Hospital, 00149 Rome, Italy; martafranco220@gmail.com; 3 Institute of Molecular Systems Biology, Department of Biology, ETH Zürich, 8093 Zurich, Switzerland; talamanca@imsb.biol.ethz.ch

**Keywords:** Long COVID, patient-reported outcomes, Fatigue Assessment Scale, severity stratification, precision medicine

## Abstract

Patient-reported outcomes (PROs) are often regarded in clinical practice as less reliable than biomarkers because they are patient-dependent and not objectively measurable in the same way as circulating molecules or imaging findings. This view may be reductive. Biomarkers are frequently considered reliable because they are easily quantifiable, yet biological meaning does not depend solely on measurement. In precision and personalized medicine, the absolute level of a circulating hormone, cytokine, or metabolite is often insufficient unless interpreted in relation to how a given patient, at a given time, responds to that molecular signal. If biomarkers are considered less absolute and more context-dependent, the relevance of PROs becomes clearer. Standardized PROs capture the subjective impact of disease on the patient system in a reproducible and clinically interpretable manner. This is particularly relevant in Long COVID, where no validated diagnostic biomarker is currently available, and severity stratification cannot rely exclusively on objective biological measures. Among available tools, the Fatigue Assessment Scale (FAS) may be particularly useful for quantifying the fatigue burden in Long COVID, including both physical and mental components. Moreover, FAS scores have been shown to correlate with the number of symptoms reported by patients, suggesting that this scale may help stratify patients not only according to fatigue severity but also according to overall symptom burden. Greater standardization of clinical characterization through instruments such as the FAS may improve comparability across cohorts and facilitate interpretation of data from different clinical trials.

## 1. Introduction

The prevalence of post-acute infection syndromes (PAISs) is increasing worldwide, establishing a massive and unprecedented challenge for global public health infrastructure [1]. Far from being a novel phenomenon unique to the SARS-CoV-2 pandemic, PAISs encompass a cluster of chronic, multi-systemic conditions triggered by diverse viral, bacterial, and parasitic pathogens [1]. Current epidemiological models estimate that approximately 10% to 20% of individuals surviving an acute viral or bacterial trigger go on to develop persistent, multi-systemic sequelae, translating to hundreds of millions of affected individuals globally [1]. Within this expanding post-infectious landscape, Long COVID represents the most visible and pervasive contemporary manifestation of this syndrome [2]. 

Long COVID was defined as a state occurring in individuals with a history of probable or confirmed SARS-CoV-2 infection that lasts for at least 2 months and cannot be explained by an alternative diagnosis [3]; more recent frameworks, such as the 2024 National Academies of Sciences, Engineering, and Medicine definition, further emphasize that individuals with symptoms persisting for at least 3 months carry a higher likelihood of continued symptoms for a year or longer [2]. This systemic condition is characterized by a broad and heterogeneous spectrum of symptoms. While it may present in a mild, self-limiting form with low impact on quality of life, it can also evolve into a chronic and highly disabling condition, with a persistent or relapsing course that substantially impairs patients’ physical, cognitive, and psychosocial functioning [4]. Beyond its clinical toll, Long COVID carries a substantial economic burden, with macroeconomic models estimating an annual global gross domestic product (GDP) loss of approximately 0.2% driven primarily by indirect costs, such as workforce absenteeism and reduced long-term productivity [5].

To date, there is no consensus definition of Long COVID, nor are validated diagnostic or prognostic biomarkers available. This lack of standardization complicates clinical management and hinders formal recognition of the condition [1]. As a result, severity stratification remains largely based on individual clinical judgment, making it difficult to objectively assess and standardize symptom burden across patients.

The absence of a shared framework for classifying Long COVID severity also limits the comparability and aggregation of data across clinical centers, thereby complicating the design and execution of multicenter studies [6,7]. Patient follow-up similarly relies on clinician-based assessments, which are inherently subjective and influenced by contextual and interpersonal factors. In a context in which chronic PAIS are becoming increasingly prevalent, the availability of a simple and practical tool capable of capturing both physical and mental disabilities is therefore of critical importance [8].

## 2. The Parallel Paradigm: Valorizing Patient-Reported Outcomes (PROs) in Conjunction with Biological Biomarkers

The current research landscape for Long COVID has identified numerous biological, radiological, and histological alterations. Soluble biomarkers, persistent spike protein reservoirs, microclots, pro-inflammatory cytokines, distinctive immunological patterns, autoantibodies, hormonal dysregulation, herpesvirus reactivations, amyloid deposits, and microbiota changes represent the primary biological correlates of the syndrome [9,10,11,12]. Nevertheless, none of these findings is pathognomonic or sufficient as a standalone diagnostic tool [13]. Consequently, patients frequently present with near-normal routine laboratory values while simultaneously experiencing profound, system-wide, and highly debilitating functional exhaustion [9]. A similar pattern is observed in mild neurocognitive impairment; patients frequently present with normal results on formal evaluations, yet they exhibit underlying deficits and manifest clear difficulties in their activities of daily living; in a large community sample, for instance, objectively measurable cognitive deficits persisted after COVID-19 even among individuals who had clinically recovered [14].

Moreover, the lack of a linear correlation between acute infection severity and subsequent Long COVID severity underscores the inadequacy of traditional clinical metrics. Since persistent, disabling sequelae frequently develop after mild or asymptomatic phases, standardized patient-reported outcomes (PROs) become indispensable, offering an important means to capture the functional burden experienced by these patients [15].

Characterizing Long COVID requires integrating objective biomarkers with standardized PROs as complementary lines of evidence. Biomarkers identify specific cellular or microscopic features, whereas PROs capture the broader functional burden on the patient. By converting subjective suffering into reproducible scores, PROs provide an essential, holistic reflection of overall disease severity alongside traditional laboratory metrics.

The conceptual elevation of PROs within precision medicine frameworks is further supported by regulatory and scientific bodies. The U.S. Food and Drug Administration (FDA) and the European Medicines Agency (EMA) have progressively recognized PROs as primary or co-primary endpoints in clinical trials, particularly in conditions where no objective surrogate exists for the patient experience [16]. In Long COVID, this recognition carries particular weight: regulatory agencies have explicitly highlighted the absence of validated surrogate endpoints and the consequent necessity of centering trial design on patient-reported functional and symptomatic outcomes [17]. This institutional endorsement legitimizes the use of instruments such as the Fatigue Assessment Scale (FAS) not merely as supplementary data collection tools but as clinically and scientifically robust primary endpoints in interventional research.

## 3. The Fatigue Assessment Scale (FAS) as an Integrative Proxy for Overall Disease Burden

Fatigue is among the most prevalent and disabling symptoms reported by individuals with Long COVID, with a substantial impact on quality of life, work capacity, and social functioning [4,9]. 

Crucially, post-acute viral fatigue cannot be reduced to mere physical exhaustion; rather, it reflects a multifaceted and significant neuropsychological burden, comprising cognitive impairment, attentional difficulties, emotional distress, and reduced stress tolerance [18,19]. Growing evidence suggests a close and potentially bidirectional relationship between fatigue severity and neuropsychological load. Within this pathogenic loop, sustained cognitive and emotional demands actively exacerbate systemic fatigue, while the fatigue itself further amplifies the patient’s perceived cognitive dysfunction and psychological distress [18,19]. In this framework, fatigue may be conceptualized as an integrative clinical construct that captures the cumulative impact of physical, cognitive, and emotional dysfunctions. Its assessment therefore can provide insight into the global burden of disease, rather than into a single symptom domain [8]. The FAS represents an ideal candidate to operationalize this integrative view. It is a brief, validated 10-item questionnaire specifically designed to assess fatigue across both physical and mental domains [20]. A total score ≥22 indicates the presence of fatigue, while a score ≥35 reflects severe fatigue. While initially validated in sarcoidosis [21], the FAS has been successfully applied across a range of chronic conditions characterized by complex symptom profiles, including autoimmune and inflammatory diseases [22] and pulmonary chronic diseases [23]. Emerging evidence confirms that the FAS is uniquely suited to capture the multidimensional symptom burden in Long COVID [24]. Recent applications of the tool in clinical and population-based cohorts support its strong psychometric validity and utility in post-COVID-19 settings [25]. Most importantly, current evidence points toward a consistent clinical correlation: higher FAS scores frequently align with an increased total symptom burden and a greater perceived severity of the patient’s overall clinical presentation [26]. These results demonstrate that the FAS is not merely a measure of tiredness but a robust proxy for Long COVID severity. By combining physical, cognitive, and neuropsychological dimensions, the instrument effectively captures the systemic nature of the syndrome.

The FAS is a brief, self-administered 10-item questionnaire; each item is scored on a five-point Likert scale, yielding a total score from 10 to 50 (with ≥22 indicating fatigue and ≥35 severe fatigue), and it typically requires only two to three minutes to complete (see Table 1). Population-based studies from Northern Europe have confirmed that FAS-derived fatigue severity predicts functional impairment across domains, including occupational performance, social participation, and health-related quality of life, independently of comorbid conditions [27]. Psychometric analyses conducted in post-COVID-19 cohorts have confirmed the instrument’s good internal consistency (Cronbach’s α of 0.89), its robust construct validity as assessed through confirmatory factor analysis, and its responsiveness to change over time, making it suitable not only for cross-sectional severity classification but also for monitoring disease trajectory and treatment response in longitudinal and interventional designs. Cross-cultural validation further supports these properties: the Japanese version of the FAS has demonstrated good reliability and validity, reinforcing the instrument’s applicability across diverse post-COVID-19 populations [28].

This is supported by evidence from our own group: in a prospective case–control study at a tertiary Long COVID clinic in Rome, patients with Long COVID (LC) scored substantially higher on the FAS than asymptomatic post-COVID (APC) controls. Median total FAS scores were 29 (IQR 22–37) in LC versus 14 (IQR 11.5–17.5) in APC (*p* < 0.001), with the same pattern across the physical (median 17, IQR 12.5–19 vs. 8.5, IQR 5.75–9.5; *p* < 0.001) and mental (median 13, IQR 9–18 vs. 6, IQR 5–7; *p* < 0.001) subdomains. Impairment of daily activities attributable to Long COVID was reported by 66 patients (24 men, 42 women) at baseline, and higher FAS scores were significantly related to both the diagnosis and the severity of the condition (*p* < 0.0001) and to worse quality of life (*p* < 0.001). Taken together, these observations position the FAS as an integrative marker of selected, highly disabling dimensions of disease burden rather than of fatigue in isolation [26].

## 4. Methodological Implications for Clinical Trials and International Standardization

In this respect, the FAS may function as a practical proxy for Long COVID severity, providing a self-reported, quantitative estimate of global disease burden that integrates physical and neuropsychological dimensions into a single, clinically meaningful score. It should be acknowledged that Long COVID is a multidimensional syndrome, encompassing cognitive impairment, dysautonomia, respiratory symptoms, pain, and sleep disturbances, and that no single self-reported scale can capture every one of its facets. Nevertheless, in our cohort, higher FAS scores were significantly associated with the diagnosis and, importantly, with the severity of Long COVID, and the scale discriminated across severity strata; this indicates that, albeit in a relatively small sample, the FAS does not merely quantify an isolated symptom but tracks the overall clinical burden of the condition. Pending the development and validation of a dedicated, concise Long COVID-specific instrument, the FAS therefore represents a pragmatic and empirically supported proxy of disease severity, while acknowledging that it should be complemented by assessments targeting the dimensions it does not directly measure. As a consequence, in our view, the FAS deserves broader consideration and systematic implementation in the context of Long COVID. The instrument is remarkably easy to administer, requires minimal completion time (usually less than two minutes), and places a negligible cognitive burden on the patient. This balance between brevity and clinical depth is particularly vital in large-scale multicenter studies and everyday clinical practice, where extensive, hours-long neurocognitive or testing batteries are frequently hindered by the patient’s severe post-exertional malaise and exhaustion [7,9]. 

Furthermore, the specific inclusion of items addressing mental fatigue enhances its capacity to capture the neuropsychological dimension of Long COVID, a critical component that is routinely underrepresented or entirely missed by purely somatic assessment scales [8]. Moreover, its availability in more than 25 languages supports cross-cultural applicability and facilitates harmonized data collection across different settings globally. Compared with other widely used fatigue instruments, such as the Chalder Fatigue Scale [29], FACIT-Fatigue [30], and PROMIS Fatigue [31], the FAS offers a favorable balance of brevity and dual (physical and mental) domain coverage, although head-to-head comparative studies in Long COVID are still lacking and would help define the most appropriate instrument for this population (Table 2).

In the context of precision and personalized medicine, particularly for emerging and only partially understood syndromes, standardized PROs should not be regarded as less reliable substitutes for objective measures but as complementary instruments that capture how a systemic disease is functionally expressed and experienced at the patient level. In Long COVID, the FAS summarizes clinically meaningful information on the global disease trajectory and functional impairment that simply cannot be inferred from laboratory parameters or radiological imaging alone. Several important avenues for future research emerge from the considerations discussed in this commentary. First, large-scale prospective studies specifically designed to validate FAS cutoffs and clinically meaningful change thresholds in Long COVID populations are warranted. While psychometric validity has been established, the minimal clinically important difference for the FAS in post-COVID contexts remains to be formally defined, a prerequisite for its rigorous use as a primary endpoint in interventional trials [32]. Second, longitudinal studies examining whether FAS trajectories over time can predict clinical recovery, relapse, or progression to more severe disability would substantially enhance the prognostic utility of the instrument. Third, the integration of FAS monitoring within digital health platforms, through validated electronic PRO (ePRO) applications, could enable real-time, low-burden, population-scale fatigue surveillance, with implications for both clinical management and health systems planning [33]. Fourth, because the FAS yields a single standardized and reproducible score, it is well suited to quantitative comparison across patient strata: systematically pairing FAS scores with demographic and clinical modifiers (e.g., age, sex, race and ethnicity, comorbid conditions, pregnancy, social determinants of health, and vaccination status at the time of infection) could help clarify how each factor shapes the risk, severity, and trajectory of Long COVID and thereby support a genuinely precision medicine approach to the syndrome.

## 5. Limitations

Several limitations should be acknowledged. First, although the FAS includes an item on being easily fatigued and therefore captures the tendency to tire readily, it was not designed to specifically measure post-exertional malaise (the worsening of symptoms after minimal physical or cognitive effort) and may consequently underestimate it; a patient with severe post-exertional malaise but comparatively low baseline fatigue could be underclassified. Second, the established cutoffs (≥22 for fatigue and ≥35 for severe fatigue) were derived in sarcoidosis and general working populations and have not yet been formally recalibrated for Long COVID, so their transferability and the minimal clinically important difference in this setting remain to be validated. Third, as a self-reported cross-sectional measure, the FAS is susceptible to recall and response biases and may not fully reflect the relapsing–remitting course of the condition. Finally, the FAS is not a patient-reported outcome measure developed specifically for Long COVID; although our data show that it captures much of the clinical complexity and severity of the syndrome, it is best positioned as a complement to, rather than a replacement for, broader multidimensional and objective assessments. In addition, fatigue is a non-specific symptom that overlaps with anxiety, depression, sleep disorders, and pre-existing chronic conditions, and possible floor or ceiling effects at the extremes of the score range have not been characterized in Long COVID; these factors should be weighed when interpreting FAS scores, even though, as noted above, higher scores track the diagnosis and severity of Long COVID in our cohort.

## 6. Conclusions

In conclusion, the FAS represents a promising and potentially useful instrument for severity assessment in Long COVID, spanning across both clinical practice and research. Wider institutional implementation of this tool could improve longitudinal patient monitoring, support severity stratification, and enhance data comparability across international cohorts. By establishing a clear, standardized, and patient-reported clinical proxy that correlates tightly with the overall symptom burden, the scientific community can bridge the gap between microscopic pathology and functional reality. Ultimately, adopting the FAS represents a crucial, non-invasive, and highly practical step toward harmonizing global clinical research and optimizing the design and endpoints of future interventional trials in Long COVID. Additional validation studies, longitudinal investigations, determination of clinically meaningful thresholds, and direct comparisons with alternative fatigue instruments remain necessary, and the development of a dedicated, streamlined score specifically designed for Long COVID would be desirable; in the meantime, however, the FAS already provides a valuable orientation for severity assessment.

## Figures and Tables

**Table 1:** The 10 items of the Fatigue Assessment Scale (FAS). Each item is rated on a five-point scale (1 = never to 5 = always); items 4 and 10 (*) are reverse-scored. Total scores range from 10 to 50, with ≥22 indicating fatigue and ≥35 indicating severe fatigue. Items 1–5 and 7–10 primarily capture physical fatigue, while items addressing mental exhaustion and concentration reflect the mental-fatigue domain.

Item	FAS statement
1	I am bothered by fatigue.
2	I get tired very quickly.
3	I don’t do much during the day.
4	I have enough energy for everyday life. *
5	Physically, I feel exhausted.
6	I have problems starting things.
7	I have problems thinking clearly.
8	I feel no desire to do anything.
9	Mentally, I feel exhausted.
10	When I am doing something, I can concentrate quite well. *

**Table 2:** Summary of representative studies reporting associations between Fatigue Assessment Scale (FAS) scores and clinical outcomes in Long COVID and related post-viral populations. Full bibliographic details are provided in the reference list.

Study (Ref)	Population/Sample	FAS Finding	Associated Outcome
Present cohort [26]	Prospective case-control: Long COVID vs. asymptomatic post-COVID controls, Rome	Higher median FAS in LC vs. APC: 29 (IQR 22–37) vs. 14 (IQR 11.5–17.5) overall; physical 17 vs. 8.5; mental 13 vs. 6 (all *p* < 0.001)	FAS related to LC diagnosis and severity (*p* < 0.0001) and to worse quality of life (*p* < 0.001); 66 patients reported daily-activity impairment
Population-based, N. Europe [27]	Post-COVID community cohort	FAS-derived fatigue severity	Predicted occupational, social, and HRQoL impairment, independent of comorbidity
Post-COVID psychometric cohort [28]	Post-COVID-19 patients (incl. Japanese validation)	Cronbach’s α 0.89; confirmed construct validity; responsive to change	Suitable for cross-sectional stratification and longitudinal monitoring
Symptom-burden analyses [6,9]	Long COVID/post-COVID fatigue cohorts	Higher FAS scores	Correlated with greater number of reported symptoms and functional limitation

## Data Availability

No new data were created or analyzed in this study. Data sharing is not applicable to this article.

## Abbreviations

COVID	Coronavirus Disease
EMA	European Medicines Agency
FAS	Fatigue Assessment Scale
FDA	Food and Drug Administration
GDP	Gross Domestic Product
PAIS	Post-Acute Infection Syndromes
PROs	Patient-Reported Outcomes

## References

[1] Choutka J., Jansari V., Hornig M., Iwasaki A. (2022). Unexplained post-acute infection syndromes. Nat. Med..

[2] National Academies of Sciences, Engineering, and Medicine, Health and Medicine Division, Board on Global Health, Board on Health Sciences Policy, Committee on Examining the Working Definition for Long COVID (2024). A Long COVID Definition: A Chronic, Systemic Disease State with Profound Consequences.

[3] Soriano J.B., Murthy S., Marshall J.C., Relan P., Diaz J.V., WHO Clinical Case Definition Working Group on Post-COVID-19 Condition (2022). A clinical case definition of post-COVID-19 condition by a Delphi consensus. Lancet Infect. Dis..

[4] Nalbandian A., Sehgal K., Gupta A., Madhavan M.V., McGroder C., Stevens J.S., Cook J.R., Nordvig A.S., Shalev D., Sehrawat T.S. (2021). Post-acute COVID-19 syndrome. Nat. Med..

[5] Cutler D.M. (2022). The Economic Cost of Long COVID: An Update.

[6] Logue J.K., Franko N.M., McCulloch D.J., McDonald D., Magedson A., Wolf C.R., Chu H.Y. (2021). Sequelae in Adults at 6 Months After COVID-19 Infection. JAMA Netw. Open..

[7] Munblit D., Nicholson T., Akrami A., Apfelbacher C., Chen J., De Groote W., Diaz J.V., Gorst S.L., Harman N., Kokorina A. (2022). A core outcome set for post-COVID-19 condition in adults for use in clinical practice and research: An international Delphi consensus study. Lancet Respir. Med..

[8] Thomas B., Pattinson R., Edwards D., Dale C., Jenkins B., Lande H., Bundy C., Davies J. (2024). Defining and measuring long COVID fatigue: A scoping review. BMJ Open..

[9] Davis H.E., McCorkell L., Vogel J.M., Topol E.J. (2023). Long COVID: Major findings, mechanisms and recommendations. Nat. Rev. Microbiol..

[10] Klein J., Wood J., Jaycox J.R., Dhodapkar R.M., Lu P., Gehlhausen J.R., Tabachnikova A., Greene K., Tabacof L., Malik A.A. (2023). Distinguishing features of long COVID identified through immune profiling. Nature.

[11] Camici M., Del Duca G., Brita A.C., Antinori A. (2024). Connecting dots of long COVID-19 pathogenesis: A vagus nerve- hypothalamic-pituitary- adrenal-mitochondrial axis dysfunction. Front. Cell. Infect. Microbiol..

[12] Pretorius E., Vlok M., Venter C., Bezuidenhout J.A., Laubscher G.J., Steenkamp J., Kell D.B. (2021). Persistent clotting protein pathology in Long COVID/Post-Acute Sequelae of COVID-19 (PASC) is accompanied by increased levels of antiplasmin. Cardiovasc. Diabetol..

[13] Thaweethai T., Jolley S.E., Karlson E.W., Levitan E.B., Levy B., McComsey G.A., McCorkell L., Nadkarni G.N., Parthasarathy S., Singh U. (2023). Development of a Definition of Postacute Sequelae of SARS-CoV-2 Infection. JAMA.

[14] Hampshire A., Azor A., Atchison C., Trender W., Hellyer P.J., Giunchiglia V., Husain M., Cooke G.S., Cooper E., Lound A. (2024). Cognition and Memory after Covid-19 in a Large Community Sample. N. Engl. J. Med..

[15] Sudre C.H., Murray B., Varsavsky T., Graham M.S., Penfold R.S., Bowyer R.C., Pujol J.C., Klaser K., Antonelli M., Canas L.S. (2021). Attributes and predictors of long COVID. Nat. Med..

[16] U.S. Food and Drug Administration (2009). Guidance for Industry: Patient-Reported Outcome Measures: Use in Medical Product Development to Support Labeling Claims.

[17] Michelen M., Manoharan L., Elkheir N., Cheng V., Dagens A., Hastie C., O’Hara M., Suett J., Dahmash D., Bugaeva P. (2021). Characterising long COVID: A living systematic review. BMJ Glob. Health.

[18] Campos M.C., Nery T., Starke A.C., de Bem Alves A.C., Speck A.E., Aguiar A.S. (2022). Post-viral fatigue in COVID-19: A review of symptom assessment methods, mental, cognitive, and physical impairment. Neurosci. Biobehav. Rev..

[19] Elboraay T., Ebada M.A., Elsayed M., Aboeldahab H.A., Salamah H.M., Rageh O., Elmallahy M., AboElfarh H.E., Mansour L.S., Nabil Y. (2025). Long-term neurological and cognitive impact of COVID-19: A systematic review and meta-analysis in over 4 million patients. BMC Neurol..

[20] Michielsen H.J., De Vries J., Van Heck G.L. (2003). Psychometric qualities of a brief self-rated fatigue measure: The Fatigue Assessment Scale. J. Psychosom. Res..

[21] De Vries J., Michielsen H., Van Heck G.L., Drent M. (2004). Measuring fatigue in sarcoidosis: The Fatigue Assessment Scale (FAS). Br. J. Health Psychol..

[22] Horisberger A., Courvoisier D., Ribi C. (2019). The Fatigue Assessment Scale as a simple and reliable tool in systemic lupus erythematosus: A cross-sectional study. Arthritis Res. Ther..

[23] Kolner-Augustson L., Prior T.S., Skivild V., Aalestrup A., Bendstrup E. (2020). Fatigue in idiopathic pulmonary fibrosis measured by the Fatigue Assessment Scale during antifibrotic treatment. Eur. Clin. Respir. J..

[24] Khatib S., Sabobeh T., Habib A., John S., Gomez R., Sivasankar S., Masoud A. (2023). Post-COVID-19 fatigue as a major health problem: A cross-sectional study from Missouri, USA. Ir. J. Med. Sci..

[25] O’Regan E., Spiliopoulos L., Bech Svalgaard I., Nielsen N.M., Vedel Sorensen A.I., Bager P., Videbech P., Ethelberg S., Koch A., Hviid A. (2024). Post-COVID-19 Condition Fatigue Outcomes Among Danish Residents. JAMA Netw. Open..

[26] Camici M., Franco M., Talamanca L., Petino M., Paulicelli J., Scarnecchia L., Ciavarella L., Orzilli T., Balducelli F., Mazzotta V. (2025). Salivary cortisol in long COVID: A marker of broader stress system and circadian rhythm dysregulation. Front. Cell. Infect. Microbiol..

[27] Poole-Wright K., Guennouni I., Sterry O., Evans R.A., Gaughran F., Chalder T. (2023). Fatigue outcomes following COVID-19: A systematic review and meta-analysis. BMJ Open..

[28] Tokumasu K., Matsuki N., Fujikawa H., Sakamoto Y., Otsuka F. (2025). Reliability and Validity of the Japanese Version of the Fatigue Assessment Scale. Intern. Med..

[29] Chalder T., Berelowitz G., Pawlikowska T., Watts L., Wessely S., Wright D., Wallace E.P. (1993). Development of a fatigue scale. J. Psychosom. Res..

[30] Yellen S.B., Cella D.F., Webster K., Blendowski C., Kaplan E. (1997). Measuring fatigue and other anemia-related symptoms with the Functional Assessment of Cancer Therapy (FACT) measurement system. J. Pain Symptom Manag..

[31] Cella D., Lai J.S., Jensen S.E., Christodoulou C., Junghaenel D.U., Reeve B.B., Stone A.A. (2016). PROMIS Fatigue Item Bank had Clinical Validity across Diverse Chronic Conditions. J. Clin. Epidemiol..

[32] Calvert M., Blazeby J., Altman D.G., Revicki D.A., Moher D., Brundage M.D., CONSORT PRO Group (2013). Reporting of patient-reported outcomes in randomized trials: The CONSORT PRO extension. JAMA.

[33] Basch E., Deal A.M., Dueck A.C., Scher H.I., Kris M.G., Hudis C., Schrag D. (2017). Overall Survival Results of a Trial Assessing Patient-Reported Outcomes for Symptom Monitoring During Routine Cancer Treatment. JAMA.

